# Graphene Oxide/Nitrocellulose Non-Covalent Hybrid as Solid Phase for Oligo-DNA Extraction from Complex Medium

**DOI:** 10.3390/molecules28124599

**Published:** 2023-06-07

**Authors:** Georgian A. Toader, Florentin R. Nitu, Mariana Ionita

**Affiliations:** 1 Faculty of Medical Engineering, University Politehnica of Bucharest, Gh Polizu 1-7, 011061 Bucharest, Romania; georgian.toader3007@upb.ro (G.A.T.); florentin.nitu@upb.ro (F.R.N.); 2 Genetic Lab, Str. Milcov, nr. 5, Sector 1, 012273 Bucuresti, Romania; 3 Advanced Polymer Materials Group, University Politehnica of Bucharest, Gheorghe Polizu 1-7, 011061 Bucharest, Romania; 4 eBio-Hub Research Centre, University Politehnica of Bucharest-Campus, Iuliu Maniu 6, 061344 Bucharest, Romania

**Keywords:** DNA extraction, graphene oxide, nitrocellulose, fluorescence quenching

## Abstract

A nitrocellulose–graphene oxide hybrid that consists of a commercially nitrocellulose (NC) membrane non-covalently modified with graphene oxide (GO) microparticles was successfully prepared for oligonucleotide extraction. The modification of NC membrane was confirmed by Fourier Transform Infrared Spectroscopy (FTIR), which highlighted the principal absorption bands of both the NC membrane at 1641, 1276, and 835 cm^−1^ (NO_2_) and of GO in the range of 3450 cm^−1^ (CH_2_-OH). The SEM analysis underlined the well-dispersed and uniform coverage of NC membrane with GO, which displayed thin spider web morphology. The wettability assay indicated that the NC–GO hybrid membrane exhibited slightly lower hydrophilic behavior, with a water contact angle of 26.7°, compared to the 15° contact angle of the NC control membrane. The NC–GO hybrid membranes were used to separate oligonucleotides that had fewer than 50 nucleotides (nt) from complex solutions. The features of the NC–GO hybrid membranes were tested for extraction periods of 30, 45, and 60 min in three different complex solutions, i.e., an aqueous medium, an α-Minimum Essential Medium (αMEM), and an αMEM supplemented with fetal bovine serum (FBS). The oligonucleotides were desorbed from the surface of the NC–GO hybrid membrane using Tris-HCl buffer with a pH of 8.0. Out of the three media utilized, the best results were achieved after 60 min incubation of the NC–GO membranes in αMEM, as evidenced by the highest fluorescence emission of 294 relative fluorescence units (r.f.u.). This value corresponded to the extraction of approximately 330–370 pg (≈7%) of the total oligo-DNA. This method is an efficient and effortless way to purify short oligonucleotides from complex solutions.

## 1. Introduction

Single-stranded deoxyribonucleic acid (ssDNA) pathogens [1], which infect organisms across all domains of life, represent a significant burden in terms of economics, medicine, and ecology [2,3]. On the other hand, in the medical field, ssDNA is emerging as a promising biomarker for a variety of illnesses, including tumors and cardiac, neurological, and infectious disorders. In order to study gene expression in diverse samples, it is essential to extract and purify nucleic acids (DNA and RNA). Various extraction methods, such as organic extraction, direct lysis extraction, and magnetic beads (MB) extraction, are used to isolate nucleic acids [4].

MB extraction has gained significant interest, as it does not utilize filters, which are specific to other methods, and it is a time-saving technique due to the faster sample collecting procedure [5]. In the standard MB extraction method, an external magnetic force is applied to draw the beads to the edge of the tube, where they trap and encapsulate the ssDNA. However, the eluted sample can become contaminated with the MB when the elution buffer is added and the magnetic field is turned off [6,7]. To address these limitations, we propose a new extraction method that combines graphenic materials [8] with NC membranes. This method utilizes the unique properties of GO to adsorb bioreceptors on its surface for the detection of various cells, molecules, ions, and nucleic acids [9,10]. The DNA immobilization on the graphenic layer can be completed by covalent and non-covalent forces, with the noncovalent π–π interaction being the major pathway [10,11,12]. ssDNA has a much higher binding efficiency on the GO surface than double-stranded (ds) DNA. There are numerous approaches where GO operates as a support nanomaterial for detecting DNA, including electrochemical [13,14], field-effect transistors [15], and fluorescent biosensor applications [16,17]. Among these, fluorescent base biosensors have been of particular interest due to their enhanced capabilities, such as low cost, simplicity, high sensitivity, superior selectivity, fast response, and multiple analyses [18]. In previous studies, GO has been utilized to retain low ssDNA concentrations through the fluorescence “turn off” quenching mechanism and as a platform for targeted ssDNA identifications in protein-containing media. This phenomenon occurs because GO can act as an electron acceptor and can efficiently quench the fluorescence of ssDNA via fluorescence resonance energy transfer (FRET) [19].

FRET takes place when the excited state energy of a fluorophore—in this case, the FAM–ssDNA—is transferred to an acceptor molecule—in this case, GO. The transfer of energy produces the quenching of the fluorescence of the ssDNA [20]. Additionally, the binding of FAM–ssDNA to GO can also result in the compaction of the FAM–ssDNA, which can further reduce the fluorescence of the FAM–ssDNA. This is because the proximity of the FAM–ssDNA to the GO can increase the probability of energy transfer through the FRET, resulting in more efficient quenching. Overall, the quenching of FAM–ssDNA by GO occurs through a combination of surface adsorption, electron transfer, and compaction effects, which result in the efficient quenching of the fluorescence of the FAM–ssDNA [21,22]. Other studies have used GO in combination with other materials, such as quantum dots and MB, to achieve better extraction of ssDNA; however, due to insufficient levels of technological development and reliable results, this approach has not proved practical in application [23].

NC is commonly used in molecular biology as a support for protein and polynucleotide binding, with a capacity of 80 µg/cm^2^. NC can also be coated with specific proteins for use in immunoassays and immobilized ssDNA for detection via hybridization. NC is also used to study the kinetics of protein–DNA interactions [24]. Because of its high electrostatic interaction with ssDNA and its ability to overcome the drawbacks of MB, NC is believed to be a suitable alternative for MB in DNA isolation [25]. Moreover, the combination of NC membrane with GO has been proposed by other studies for micro-RNA (miRNA) [26] and circulating free DNA (cfDNA) molecules’ [27] extraction.

In our approach, we have developed a novel hybrid membrane platform by combining the properties of NC and GO to enhance the immobilization of short oligonucleotide sequences in complex media. The successful coating of the cellulose nitrate membrane with GO dispersion was demonstrated through scanning electron microscopy (SEM) and Fourier transform infrared (FT-IR) spectroscopy [28]. 

The NC–GO hybrid membrane extraction features were tested using fluorescent-marked ssDNA samples in water, αMEM, and αMEM supplemented with FBS for extraction periods of 30, 45, and 60 min. The fluorescence-marked ssDNA was then detected by incubating the membranes in a desorption solution, releasing the oligonucleotides from the NC–GO hybrid membrane in a solution suitable for fluorescence analysis. Overall, our new platform offers a powerful tool for the isolation of oligonucleotides in various media.

## 2. Results and Discussion

### 2.1. Characterization of NC–GO Hybrid Membrane Array

#### 2.1.1. FTIR Spectra

**Figure 1:**
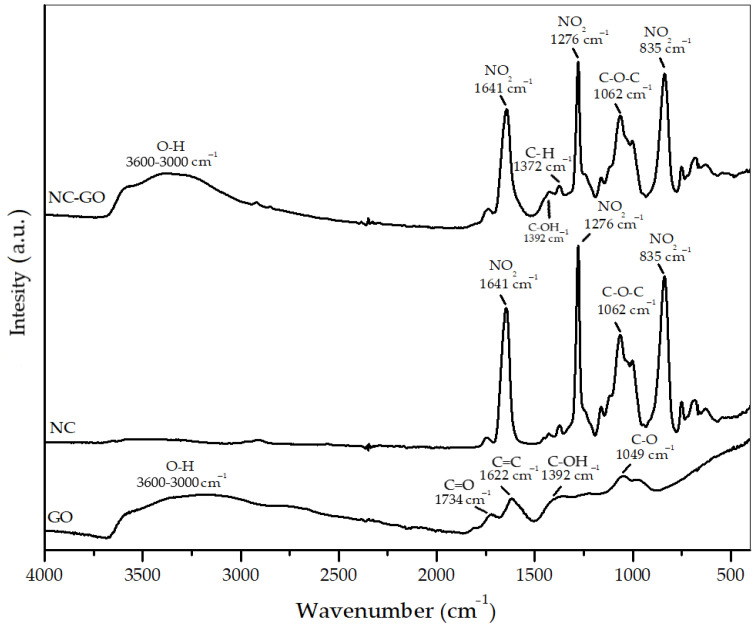
FTIR spectra of GO, NC, and NC–GO hybrid membrane.

Figure 1 presents the FTIR spectra obtained for GO, NC membrane, and NC–GO hybrid membrane. The GO typical spectrum is characterized by absorption bands located around 1040–1050, 1330–1420, 1600–1620, and 1735 cm^−1^ [29,30]. These bands correspond to the vibrational modes of different groups, such as C–O (alkoxy/epoxy), C–OH (carboxy), C=C (aromatics), and C=O (carbonyl), respectively. Additionally, the presence of O–H (hydroxyl) stretching vibrations, both in their free and intermolecular bonds, are indicated by a band that extends around 3450 cm^−1^. In our specific case, the absorption bands mentioned above are observed at 1049, 1392, 1622, and 1734 cm^−1^, and in the range of 3000 to 3600 cm^−1^. NC FTIR spectrum displays three main absorption peaks: two sharp and intense peaks at approximately 1641 cm^−1^ and 1276 cm^−1^, corresponding to the asymmetric and symmetric stretching of the NO_2_ group, respectively, along with a slightly broader and more intense peak around 835 cm^−1^, assigned to the O–NO_2_ stretching [31,32]. Furthermore, the absorption band observed at 1062 cm^−1^ indicates the presence of epoxy groups (C–O–C), specific to NC. 

The FTIR spectrum of the NC–GO hybrid membrane reveals distinctive vibrational modes that correspond to both GO and NC, indicating the successful formation of NC–GO membrane. The presence of GO is evident from the broad band corresponding to O-H stretching vibrations observed at 3500 cm^−1^ together with the absorption peak at 1392 cm^−1^, matching the C–OH bending vibrations. However, certain absorption bands within the range of 830 to 1660 cm^−1^ are common for the two components and, very likely, they are overlapping.

The formation of the NC–GO hybrid membrane presents numerous advantages. GO possesses a large surface area and functional groups that can interact with biomolecules, while NC provides structural support and stability. This synergistic effect ensures that the hybrid membrane can withstand mechanical stressors, such as bending or stretching, without compromising its integrity. The combination of GO and NC also enables the modification of the hybrid membrane’s surface properties. The presence of NC within the GO matrix enhances the accessibility of adsorption sites, leading to more effective binding and immobilization of molecules [33,34,35].

#### 2.1.2. Morphological Characterization

**Figure 2:**
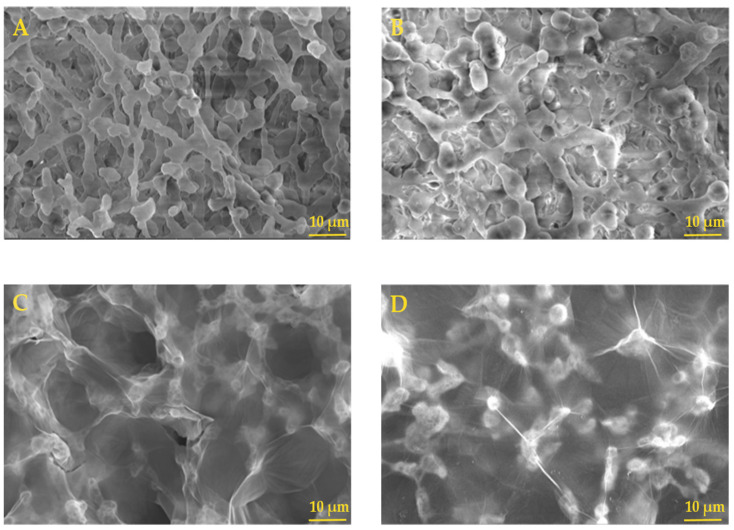
SEM images of NC membrane before the experiment (**A**), NC membrane after the experiment (**B**), NC–GO membrane before the experiment (**C**), NC–GO after the experiment (**D**); 10 μm scale bar.

The NC and NC–GO hybrid membranes were examined before and after the adsorption procedure. The SEM images in Figure 2A revealed that the NC membranes had a porous and rough surface before the experiment. After incubating the NC membrane in a complex solution, the membrane appeared less porous and less rough, probably due to the interaction with water molecules and adsorption of the of the FAM–ssDNA probe (Figure 2B). This observation suggested that the membrane was efficient in immobilizing the FAM–ssDNA, which covered the surface and clogged the membrane pores, resulting in a more uniform surface morphology. Adding GO to the surface of NC membranes had a significant impact on the surface morphology of the resulting hybrid membrane.

The SEM images in Figure 2C showed that the hybrid membrane had a morphology surface similar to a thin spider web, which covered all the NC structure’s pores and roughness. The thin layer of GO can be seen as a transparent veil beneath which the NC structure can be observed. This new surface morphology is particularly interesting because it shows uniform coverage and excellent GO dispersion. It is believed that GO contains fewer than three layers, considering the GO transparency.

The SEM images in Figure 2D showed that the hybrid membranes underwent changes in their morphology when they were exposed to working media; however, this was less pronounced than in the case of pure NC membrane. This was due to the immobilization of the FAM–ssDNA on the membrane, which resulted in a more loaded and opaque morphology when compared to the NC–GO membrane before the experiment. The homogeneous coverage with FAM–ssDNA could be attributed to the chemical groups such as carboxyl acid, hydroxyl, epoxide, and carbonyl from the GO structure, and also to π–π interaction, which provides more binding sites for attaching the FAM–ssDNA probe. Furthermore, by comparing the surface morphology of NC and NC–GO after the experiment, their structures appeared dissimilar, with rather narrow clogged or collapsed pores for pure NC and well-preserved morphology for the NC–GO hybrid membrane. This observation is an indication that GO modification of the NC membrane enhances stability in different complex solutions. 

#### 2.1.3. Wettability Properties

**Figure 3:**
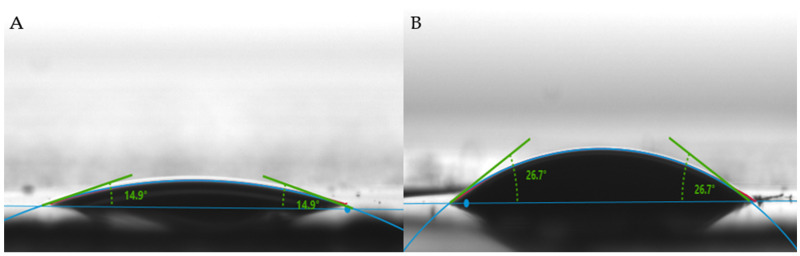
Water contact angle of (**A**) NC and (**B**) NC–GO hybrid membrane.

Figure 3 exhibits the wettability characteristics of the NC and NC–GO hybrid membranes, measured by the water contact angle. The results indicate that the surface of both membranes exhibit hydrophilic characteristics. For the NC membrane (Figure 3A), the water contact angles are closer to 15°. The water droplet spreads out significantly over the surface, indicating that the membrane has good wetting properties and tends to attract and absorb water. 

The NC–GO hybrid membrane (Figure 3B) has contact angles closer to 26°. This larger contact angle indicates that the surface of the membrane is less hydrophilic compared to the NC membrane. The water droplet spread out less on this surface, suggesting that the membrane has a reduced affinity for water. The addition of GO onto the NC membrane alters its surface properties by decreasing its hydrophilicity [36].

The surface features of the NC–GO hybrid membrane play an important role in how biomolecules interact with the membrane. Biomolecules such as ssDNA possess hydrophobic regions within their structure, primarily arising from the nonpolar bases (adenine, cytosine, guanine, and thymine) and the hydrophilic sugar–phosphate that are attracted to the hydrophilic sites of the NC–GO hybrid. The cumulative effect of hydrogen bonding and electrostatic interactions leads to increased stability and stronger affinity between ssDNA and the NC–GO membrane [37,38,39].

### 2.2. Assay Conditions Influence FAM–ssDNA Fluorescence: Detection and Extraction of Oligo DNA Using NC–GO Hybrid Membranes in Complex Media

**Figure 4:**
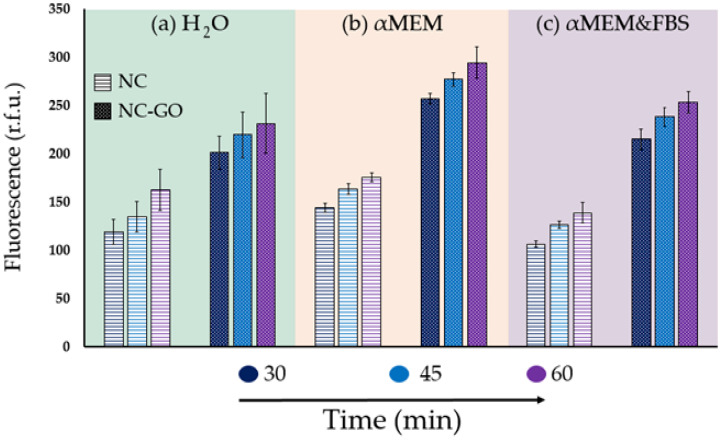
Fluorescent signal of the desorbed FAM–ssDNA after incubation of NC and NC–GO membranes in the (**a**) H_2_O, (**b**) αMEM, (**c**) αMEM + FBS media for 30, 45, and 60 min.

To examine how different assay conditions of H_2_O, αMEM, and αMEM + FBS media affect the NC and NC–GO hybrid membranes’ performance, we measured the fluorescence after incubating for 30, 45, and 60 min in the dark. The recorded results were then plotted in Figure 4. The first set of results from Figure 4a showed that the NC–GO hybrid membrane can detect a significantly higher amount of oligo DNA compared to NC membranes when incubated in water. The fluorescence values for NC–GO membranes were approximately 100 r.f.u. higher than those of control membranes at all incubation times, indicating that GO provides higher affinity and anchoring sites with more spots for oligonucleotide extraction. The incorporation of GO sheets improves the NC membrane’s ability to extract oligo ssDNA, owing to the distinct physical and chemical characteristics of GO. Furthermore, the large surface area and high aspect ratio of GO sheets provide a greater number of binding sites and more exposure to the functional groups, further enhancing their ability to extract oligo ssDNA. Therefore, the addition of GO to NC membranes can significantly increase their capacity for oligo ssDNA immobilization, leading to a higher amount of extracted oligo ssDNA and a stronger fluorescence signal [40,41,42,43].

Another key factor in the adsorption and desorption kinetics of ssDNA from NC–GO hybrid membrane is the pH of the solution. The pH influences the electrostatic interactions and hydrogen bonding between the ssDNA and the hybrid membrane, thus affecting the adsorption and desorption processes. At pH 7.0, in the presence of salts, the interaction between GO and ssDNA is facilitated by the Na^+^ positively charged ions that can fence in the negative charges from the GO surface [44].

Conversely, at pH 8.0, when the solution has low ionic strength or no salts, the repulsive electrostatic forces between the negatively charged GO surface and ssDNA dominate. As a result, the ssDNA molecules tend to detach or desorb from the GO surface and spread in the solution [45,46,47,48].

The best results for fluorescence were obtained when the membranes were incubated in the αMEM medium. Figure 4b indicates that these NC and NC–GO hybrid membranes have a much stronger effect in terms of interaction with oligo ssDNA molecules than those from the H_2_O medium. The composition of αMEM used for incubation enhances oligo ssDNA adsorption due to the presence of various components that could improve the binding of oligo ssDNA to the membrane surface. αMEM contains calcium and magnesium ions, which help stabilize the oligo ssDNA structure and facilitate its interaction with the functional groups on GO. The presence of these ions can also help to induce a flow of water from the surrounding medium into the NC–GO hybrid membrane, which could help to promote the adsorption of oligo ssDNA onto the membrane surface.

Additionally, the presence of serum proteins in αMEM, such as BSA, may help to reduce the non-specific binding of oligo ssDNA to other surfaces in the medium and enhance its binding to the membrane. BSA shields the negatively charged oligo ssDNA molecules from repulsive electrostatic interactions with GO. Moreover, αMEM contains glucose and other carbohydrates that can increase the osmotic pressure of the solution. The increase in osmotic pressure could induce a flow of water from the surrounding medium into the membrane, which could promote the adsorption of oligo ssDNA onto the membrane surface [49,50,51].

The third medium investigated was based on αMEM supplemented with 10% FBS and was meant to simulate human plasma. The results shown in Figure 4c indicate that both types of membranes had a lower fluorescence effect when incubated in the current αMEM + FBS medium compared to the previous αMEM medium. This is mainly attributed to the incorporation of FBS, which blocks the fluorescence emission. 

FBS is a commonly used supplement in cell culture media that provides necessary nutrients and growth factors to support cell growth and proliferation. However, FBS contains many proteins that can potentially interfere with fluorescence-based assays. One major problem is that FBS contains endogenous fluorophores, such as nicotinamide adenine dinucleotide (NAD), and riboflavin, which can emit fluorescence and interfere with the signals of exogenous fluorescent dyes or proteins used by researchers to study cellular processes. This can result in high background fluorescence, making it difficult to detect and quantify the fluorescence signal of interest. Additionally, FBS can contain other compounds, such as quenchers or inhibitors, that can affect the fluorescence signal by either reducing the intensity or altering the spectral properties of the fluorophore [52,53,54,55,56,57,58]. 

Even though the fluorescence effect is decreased in the desorption solution, it is important to mention that NC–GO membranes have a greater ability to extract oligo ssDNA over time than NC control membranes. This is evident from the fluorescence signal of NC–GO membranes, which have values above 200 r.f.u., indicating significantly higher oligo ssDNA adsorption compared to NC control membranes, which have values above 150 r.f.u. 

Aspects related to FBS interference in fluorescence measurements are presented in Table 1. The fluorescence of the media was recorded both before and after the addition of the FAM–ssDNA probe. The recorded αMEM media fluorescence was 1685 r.f.u. Upon the addition of FBS, the fluorescence of the media increased to 2016 r.f.u.

Furthermore, upon addition of the FAM–ssDNA probe, the fluorescence of both samples further increased to 5493 and 5504 r.f.u. The difference between the fluorescence readings before and after adding the FAM–ssDNA probe indicates that FBS reduces the fluorescence signal of the media. 

To ensure the accuracy of the fluorescent signal of FAM–ssDNA in the desorption solution, we conducted tests by incubating the NC–GO hybrid membranes in αMEM and αMEM + FBS media without FAM–ssDNA. The results, as shown in Table 1, indicate that, in the desorption solution, there were no detectable molecules from the media. This could be attributed to the inclusion of SDS in media that inactivates nucleases and controls non-specific adsorption to the NC–GO hybrid membrane surface [59,60].

Table 2 presents the mass (pg) and standard deviation of oligo ssDNA desorbed from NC and NC–GO hybrid membranes at various adsorption times and incubation media. The results suggest that the amount of FAM–ssDNA adsorbed on NC and NC–GO membranes depends on the incubation medium and the duration of the adsorption process. As the adsorption time increases, more FAM–ssDNA molecules bind to the NC and NC–GO membranes over time. Furthermore, the amount of FAM–ssDNA adsorbed in the αMEM medium is higher than in H_2_O and αMEM + FBS adsorption media, indicating that αMEM is a better incubation medium for adsorbing FAM–ssDNA on NC and NC–GO hybrid membranes.

Comparing the total amount of oligo ssDNA at different adsorption times within the same extraction medium provides valuable information on the binding kinetics of oligo DNA molecules onto the adsorbent material. For instance, in the case of NC membranes, the highest amount of mass desorbed was observed after 60 min in αMEM (205 pg, ±5 s.d.), while the lowest desorption was observed after 30 min in αMEM + FBS (124.3 pg, ±3.4 s.d.). Similarly, for NC–GO membranes, the highest amount of oligo ssDNA desorbed was observed after 60 min in αMEM (343.6 pg, ±19.1 s.d.), while the lowest mass was observed after 30 min in αMEM + FBS (251 pg, ±10.8 s.d.). It is relevant to highlight that the amount of oligo ssDNA desorbed from the NC–GO membrane is generally higher than that from the NC membrane. 

While the membrane successfully extracts a significant amount of DNA from the media, it falls short in comparison to commercially available kits. For instance, the MGIEasy magnetic beads extraction kit [61] demonstrates a DNA extraction range of 71 to 74 ng/µL from a human blood sample with an elution volume of 100 µL, surpassing our highest quantity of 343.6 pg obtained in αMEM. Because our technique extracts a lower amount of DNA compared to a widely used kit, we acknowledge that the need for improvement has to be explored. Conducting additional studies will enable us to improve the efficiency and reliability of our technique, making it more versatile for extracting various types of biomolecules from different types of samples, contributing to molecular biology applications and other fields that rely on accurate biomolecule extraction.

## 3. Materials and Methods

### 3.1. Reagents

The GO dispersion, cat. No. 763705, of 2 mg/mL concentration in water with mean layer diameter of less than 10 μm and a composition of 42–52% carbon and 44–45% oxygen, was procured from Sigma-Aldrich (St. Louis, MO, USA). The bovine serum albumin (BSA), sodium chloride (NaCl), Trizma^®^ hydrochloride-99% (Tris-HCl), and sodium dodecyl sulfate (SDS), having the formula CH_3_(CH_2_)_11_OSO_3_Na, were acquired from Sigma-Aldrich (St. Louis, MO, USA). NC membranes, type 11301, with 8.0 µm pore size and a 47 mm diameter, were purchased from Sartorius (Gottingen, Niedersachsen, Germany). The ssDNA oligonucleotides were acquired from Integrated DNA Technologies, Inc. (Coralville, IA, USA). The used ssDNA oligonucleotide sequences consisted of 5′-TTT CAA CAT CAG TCT GAT AAG CTA TCT CCC-3′ and were labeled at the last primer with 6-fluorescein amidite (6-FAM), forming the complex known as FAM–ssDNA. The αMEM, M4526, and 10% Fetal Bovine Serum, F7524, were bought from Sigma-Aldrich (St. Louis, MO, USA).

### 3.2. NC–GO Hybrid Membrane Preparation

**Figure 5:**
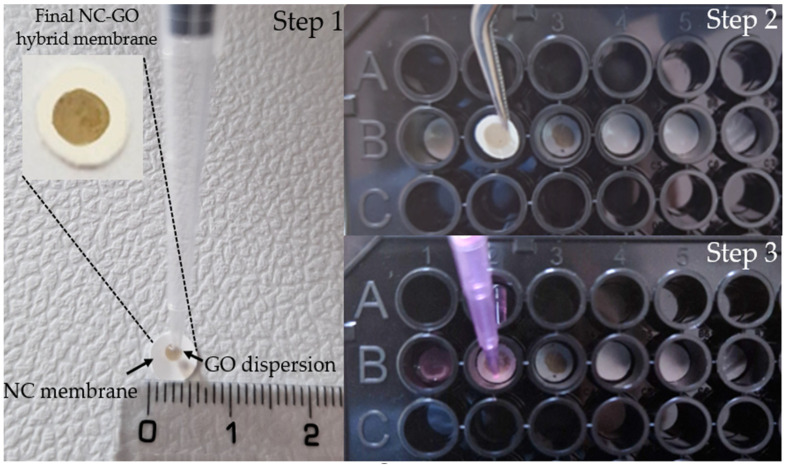
(Step 1) Fabrication of NC–GO hybrid membrane by drop casting a dispersion of GO onto the surface of NC membrane; (Step 2) Membrane insertion in the Costar 96-well flat-bottomed plates; (Step 3) Flooding of membranes with solutions.

The NC membranes with a diameter of 5 mm and ~1.1 mg weight were cut by using a paper puncher. Furthermore, for the non-covalent modification of the NC membrane, we used a technique similar to the dot blot method [62]. GO dilution in deionized water from 2 mg/mL GO to 400 µg/mL GO was first performed. After that, 5 µL of GO diluted dispersion was applied onto each NC disc by drop casting (Step 1 from Figure 5). The NC–GO hybrid membranes were air-dried overnight and washed with DI water prior to use.

### 3.3. Preparation and Desorption Procedure of FAM–ssDNA from NC and NC–GO Hybrid Membranes

Firstly, three different complex solutions, water, αMEM, and αMEM + FBS, were prepared. Each of these media contained 16 nM of FAM–ssDNA, 0.1% SDS, 0.1 mg/mL BSA, 100 mM NaCl, and 10 mM Tris-HCl. pH 7.0 SDS surfactant was used to maintain the GO dispersion and to inactivate enzymes such as DNases from the cell culture medium. As Figure 5 shows, the samples were spread into black Costar 96-well flat-bottomed plates with a net capacity of 100 µL per well. 

To begin the adsorption procedure, a total of six membranes were used, three NC membranes and three NC–GO hybrid membranes (Step 2 from Figure 5). Each of these membranes was incubated in three different media for 30, 45, and 60 min to allow for FAM–ssDNA binding (Step 3 from Figure 5). 

After incubation, the membranes were washed in water to remove unbounded oligo ssDNA and then placed in a 10 mM Tris-HCl pH 8.0 solution for 45 min for the FAM–ssDNA desorption procedure. After removing the NC–GO discs, the fluorescence of the solution containing the desorbed FAM–DNA was measured at 535 nm using a microplate reader. The data obtained from the measurements were converted into weight units utilizing Equation (1) and are reported in Table 2.

(1) $$M_{f}=\frac{F_{f}*M_{i}}{F_{i}}$$

where *M_f_* is the final mass of the desorbed FAM–ssDNA in pg; *F_f_* is the final fluorescence in r.f.u. measured after the desorption process; *M_i_* is the initial mass of the FAM–ssDNA (≈4420 pg) obtained by using the “DNA molecular weight and conversion” tools from ThermoFisher [63]; and *F_i_* represents the fluorescence in r.f.u. of the solution after addition of the FAM–ssDNA sequence.

### 3.4. Spectroflourimeter Assay

The intensity of emitted fluorescence was measured using a TECAN Spark Fluorescence microplate reader (Tecan Trading AG., Männedorf, Switzerland) at 535 nm with 5 readings recorded for every well. The adsorption kinetics were monitored at 23 °C using the same microplate reader.

### 3.5. Membrane Characterization

To examine the interactions between NC and GO, we conducted FTIR studies using a SHIMADZU 8900 piece of equipment (Kyoto, Japan). The FTIR spectra were recorded over a range of 400–4000 cm^−1^ with a resolution of 4 cm^−1^, averaging 32 measurements per sample.

FEI’s Quanta F 250 scanning electron microscope was used to examine the surface morphology of the NC membranes before and after they were coated with GO particles, as well as before and after the hybrid was used in experiments. Prior to the SEM investigation, the samples were coated with a thin layer of gold–palladium to enhance their conductivity. 

Information about the membranes’ wettability was obtained using the Drop Shape Analyzer-DSA100 from Krüss Scientific GmbH (Hamburg, Germany) and the sessile drop method. The effect of GO on the wettability of NC membranes was evaluated by static water contact angle measurements at room temperature. The shape of the deionized water drop on the sample surface was recorded with a CF03 digital camera for 5 s after the deposition of the droplet with a volume of 2 μL. The values of the water contact angle were determined using the DSA3software and represented the average of three measurements for each sample. The results were revealed using the Young–Laplace equation. 

## 4. Conclusions

We propose the use of NC–GO hybrid membranes as a promising alternative or complementary approach to commercially available magnetic particle kits for oligonucleotide extraction. The successful fabrication of the NC–GO hybrid membrane was confirmed by the FTIR spectra and SEM investigation method. The SEM micrographs illustrated a uniform coverage and good dispersion of GO onto the NC membrane surface in a thin spider web morphology. The wettability properties showed that both membranes were in the hydrophilic range, but the NC–GO hybrid membrane exerted a more hydrophobic water contact angle at 26.7° than the NC control membrane, which exhibited a 15° contact angle. 

The current results indicate that the NC–GO hybrid membrane can be used as non-covalent hybrid solid phase capable of detecting, extracting, and desorbing oligo-DNA in H_2_O, αMEM, and αMEM + FBS. Among them, αMEM proved to be the most effective.

Additionally, our study showed that NC membranes have a limited capacity for immobilizing oligo ssDNA, with the lowest fluorescent signal of 106.5 r.f.u. being attributed to a desorbed mass of 124.3 pg after 30 min of incubation in αMEM + FBS. The results indicate that modifying NC membranes with GO greatly enhances their interaction with oligo ssDNA, leading to much higher binding affinity. Specifically, after 60 min of FAM–ssDNA adsorption in αMEM, the highest fluorescent signal of 294.4 r.f.u. was observed, corresponding to an oligo ssDNA quantity of 343.6 pg.

Moreover, the fluorescence values increased with the incubation time for both membranes, indicating that ssDNA extraction from complex media is a time-dependent process. Importantly, NC–GO hybrid membranes extracted more ssDNA than NC membranes, regardless of the incubation time and media used.

Therefore, we conclude that NC–GO hybrid membranes can serve as an alternative or supplementary technique for MB extraction kits, presenting essential characteristics such as reproducibility, low cost, and rapid assay response. 

## Figures and Tables

**Table 1:** Average fluorescence intensity of the media before and after adding the FAM–ssDNA probe, and fluorescent signal of the desorption solution after incubation of the NC–GO hybrid membrane in αMEM and αMEM + FBS media without FAM–ssDNA.

Fluorescent Signal before Adding FAM–ssDNA Sample			Fluorescent Signal after Adding FAM–ssDNA Sample			Fluorescent Signal of the Desorption Solution after Incubation of NC–GO Hybrid Membrane without FAM–ssDNA	
H_2_O	αMEM	αMEM + FBS	H_2_O	αMEM	αMEM + FBS	αMEM	αMEM + FBS
Average fluorescent signal (r.f.u.)							
68.2 ± 8.1	1685.6 ± 209.6	2016.6 ± 231.7	3796.8 ± 486.5	5493.0 ± 785.4	5504.2 ± 764.3	74.7 ± 8.5	73.9 ± 10.3

**Table 2:** Mass (pg) and standard deviation (s.d.) of the oligo ssDNA desorbed from the NC and NC–GO hybrid membrane at different incubation times.

Incubation Media	Mass (pg) and Standard Deviation of Oligo ssDNA Desorbed from NC Membrane at Different Adsorption Times						Mass (pg) and Standard Deviation of Oligo ssDNA Desorbed from NC–GO Hybrid Membrane at Different Adsorption Times					
Time	30 min		45 min		60 min		30 min		45 min		60 min	
U/M	pg	s.d.	pg	s.d.	pg	s.d.	pg	s.d.	pg	s.d.	pg	s.d.
H_2_O	139.1	±15.4	157.5	±18.2	189.4	±24.7	234.8	±20.1	256.4	±27.6	269.9	±36.3
αMEM	168.2	±5.4	191.2	±6.3	205.0	±5.0	299.8	±6.1	323.4	±8.0	343.6	±19.1
αMEM + FBS	124.3	±3.9	147.5	±4.8	162.1	±12.6	251.1	±12.3	278.2	±11.2	296.2	±12.9

U/M means Unit of Measurement.

## Data Availability

Not applicable.
